# Bioinformatics analysis of gene expression profile and functional analysis in periodontitis and Parkinson’s disease

**DOI:** 10.3389/fnagi.2022.1029637

**Published:** 2022-11-10

**Authors:** Xiaofeng Wang, Naixu Shi, Baiao Wu, Lin Yuan, Jiapeng Chen, Cong Ye, Miao Hao

**Affiliations:** ^1^Department of Stomatology, China–Japan Union Hospital of Jilin University, Changchun, China; ^2^Key Laboratory of Lymphatic Surgery Jilin Province, Jilin Engineering Laboratory for Lymphatic Surgery, China-Japan Union Hospital of Jilin University, Changchun, China; ^3^Oral and Maxillofacial Surgery, Changchun Stomatological Hospital, Changchun, China; ^4^Department of Gynecology and Obstetrics, China-Japan Union Hospital of Jilin University, Changchun, China; ^5^Department of Pathophysiology, College of Basic Medical Sciences, Jilin University, Changchun, China; ^6^Scientific Research Center, China-Japan Union Hospital of Jilin University, Changchun, China

**Keywords:** periodontitis, Parkinsion’s disease, protein–protein network interaction, differentially expressed genes, bioinformatics

## Abstract

Periodontitis is a chronic inflammatory disease inextricably linked to both the innate and acquired immune systems of the body. Parkinson’s disease (PD) is a neurodegenerative disease caused by immune system dysfunction. Although recent studies suggest that a clinical relationship exists between PD and periodontitis, the pathogenesis of this relationship is unclear. Therefore, in the present study, we obtained datasets of periodontitis and PD from the Gene Expression Omnibus (GEO) database and extracted 785 differentially expressed genes (DEGs), including 15 common upregulated genes and four common downregulated genes. We performed enrichment analyses of these DEGs using Gene Ontology (GO) and Kyoto Encyclopedia of Genes and Genomes analyses. We found that the genes were mainly enriched in keratinocyte differentiation, neuronal cell bodies, and structural constituents of epidermis terms, and pathways such as immune response and synaptic pathways. In addition, we screened matching hub genes by constructing a protein–protein interaction (PPI) network map and a Molecular Complex Detection (MCODE) map using the Cytoscape software. The hub genes were then subjected to GO enrichment analysis, which revealed that the dopamine biosynthetic process, dopaminergic synapse and dopamine-binding terms, and dopaminergic synapse and serotonergic synapse pathways were primarily where they were expressed. Finally, we selected four of these genes for validation in the periodontitis and PD datasets, and we confirmed that these hub genes were highly sensitive and specific for diagnosing and monitoring PD and periodontitis. In conclusion, the above experimental results indicate that periodontitis is a high-risk factor for PD, and the association between these two conditions is mainly manifested in immune and dopamine-related pathways. Hub genes, such as the *CDSN*, *TH*, *DDC*, and *SLC6A3* genes, may serve as potential biomarkers for diagnosing or detecting PD.

## Introduction

Periodontitis is an infectious, multifactorial condition that damages supportive tissues of the teeth (Corrêa et al., 2017). As one of the most prevalent inflammatory diseases in the world, periodontitis is not only associated with the enhancement of multiple inflammatory conditions, such as cardiovascular disease and rheumatoid arthritis (Dutzan et al., 2017), but also leads to a variety of chronic diseases, such as hypertension (Czesnikiewicz-Guzik et al., 2019) and diabetes (Xiao et al., 2017). *Porphyromonas gingivalis* (*P. gingivalis*) is the main oral pathogen responsible for the onset and progression of periodontitis (Xu et al., 2016; Ye et al., 2017). Many reports have suggested that *P. gingivalis* infection is strongly associated with Alzheimer’s disease (Liang et al., 2020).

Neurodegenerative diseases are characterized by progressive loss of neurons and neuronal death, such as Alzheimer’s disease (AD), Parkinson’s disease (PD), and Huntington’s disease (HD) (Hashimoto et al., 2019). Currently, an increasing number of studies have been suggesting the existence of an association between neurodegenerative disease and periodontitis. For example, lipopolysaccharides (LPS) from *P. gingivalis* upregulate the expression of amyloid precursor protein (APP) in the brain, promote the deposition of Aβ in neurons, and promote the development and progression of AD (Zeng et al., 2021). Moreover, a recent study using bioinformatics analysis identified a series of potential key genes, along with enriched signaling cascades, serving as promising molecular markers for periodontitis and AD (Jiang et al., 2021).

Similarly, PD is the second most common neurodegenerative disorder after AD (Kern et al., 2021). Some studies have suggested that periodontitis may lead to the occurrence and development of PD. For instance, periodontitis has been associated with changes in leukocyte counts in patients with PD (Botelho et al., 2020a). Moreover, a recent study revealed that major virulence factors of *P. gingivalis*, such as gingipain R1 (RgpA) and lipopolysaccharide, circulate in the blood of PD patients (Olsen et al., 2020). Nonetheless, researchers constructed a network of potential protein–protein interactions (PPI) between PD and periodontitis using preliminary bioinformatics analyses, in an attempt to identify new potential targets (Botelho et al., 2020b). The potential biomarkers of periodontitis-related PD and their underlying molecular mechanisms still remain poorly understood.

Recently, high-throughput sequencing has been widely used for prognostic evaluation, molecular diagnosis, and target discovery (Field, 2021). The Gene Expression Omnibus (GEO) database has huge disease gene expression patterns and a wide range of key genes that can be utilized to explore disease initiation and progression (Patra et al., 2020). The GEO database can be also used to explore the correlation between pathogenesis and molecular mechanisms, which have important clinical significance in disease research (Alameer and Chicco, 2021). Therefore, we attempted to use the GEO expression profile to identify novel biomarkers for diagnosis and therapy.

**Figure 1:**
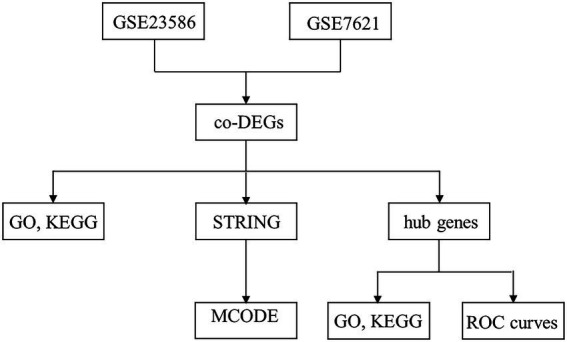
Research flow chart.

In the present study, we obtained the expression patterns of human PD and periodontitis genes from the GEO database. Using the R package (V. 3.6.3), differentially expressed genes (DEGs) were screened and the common genes were intersected. The DEGs were then used for gene ontology (GO) enrichment and Kyoto Encyclopedia of Genes and Genomes (KEGG) pathway analyses. The Search Tool for the Retrieval of Interacting Genes (STRING) was used to identify the PPI network between periodontitis and PD. Using the Cytoscape software, highly related functional modules and candidate hub genes were screened. Finally, the diagnostic values of the hub genes were determined using different independent periodontitis and PD cohorts. Summarily, in the present study, we provide promising biomarkers that might be associated with PD, and we explore the molecular mechanisms underlying PD. Our findings provide evidence for the correlation between periodontitis and PD, and provide information that could be helpful in the diagnosis and treatment of patients with PD. The article research process is shown in Figure 1.

## Materials and methods

### Data abstraction

We downloaded the gene expression array data, GSE23586 and GSE7621, from the Gene Expression Omnibus (GEO).^1^ TheGSE23586 dataset contains data obtained from the gingival tissues from three healthy subjects and three patients affected by periodontitis. Contrarily, the GSE7621 dataset contains data obtained from nine substantia nigra tissues from postmortem brain specimens of normal patients and 16 substantia nigra tissues from postmortem brain specimens of patients with PD.

### Identification of differentially expressed genes

To identify DEGs in the GSE23586 and GSE7621 datasets, the original files downloaded from the GEO database were processed and normalized using the R package (version 3.6.3; Edgar et al., 2002; Barrett et al., 2013). A|logFC| > 2 (Fold change) and *p* < 0.05 were considered the threshold for the DEGs of GSE23586. However, a|logFC| > 1 and *p* < 0.05 were considered the threshold for the DEGs of GSE7621. Genes with *p* < 0.05 and log FC > 1 were considered upregulated (UP), while genes with *p* < 0.05 and log FC < −1 were considered downregulated (DOWN). After utilizing these screening conditions, two sets of DEGs were identified, which were then put into the online Venn diagram analysis tool^2^ to obtain up- and downregulated intersection genes. To better visualize these DEGs, heat maps and volcano plots were made produced using the R package. These intersecting (common) genes were used in the subsequent analyses.

### Functional enrichment analysis of DEGs

Gene Ontology (GO) terms (for biological processes, cellular components, and molecular functions categories), and KEGG functional enrichment were performed on the intersecting DEGs using the R package.

### Protein–protein interaction network construction and module analysis

To construct a PPI association between periodontitis and PD, we used the Search Tool for the Retrieval of Interacting Genes (STRING) 11.0 (Szklarczyk et al., 2015).^3^ An interaction with a combined score > 0.7 was selected, and the PPI network was constructed using the Cytoscape software. Cytoscape (version 3.9.1) is an open-source bioinformatics software platform for visualizing networks of molecular interactions (Szklarczyk et al., 2019). Analysis of densely connected regions was performed using the Cytoscape plug-in molecular complex detection (MCODE), and the top 15 genes with the greatest relevance were screened. GO and KEGG analyses were performed using the R package.

### Statistical analyses

As previously stated, statistical analyses of all data were performed using the R software (version 3.6.3). Statistical significance was set at *p* < 0.05. Using the Statistical Packages for the Social Sciences software (SPSS 22.0; SPSS, Inc., Chicago, IL, United States), we constructed receiver operating characteristic (ROC) curves and calculated the areas under the curve (AUC) of the hub gene. We compared AUC as an indicator of the models. These results demonstrated the diagnostic efficiency of these genes. Statistical significance was set at *p* < 0.05.

## Results

### DEGs identification

**Figure 2:**
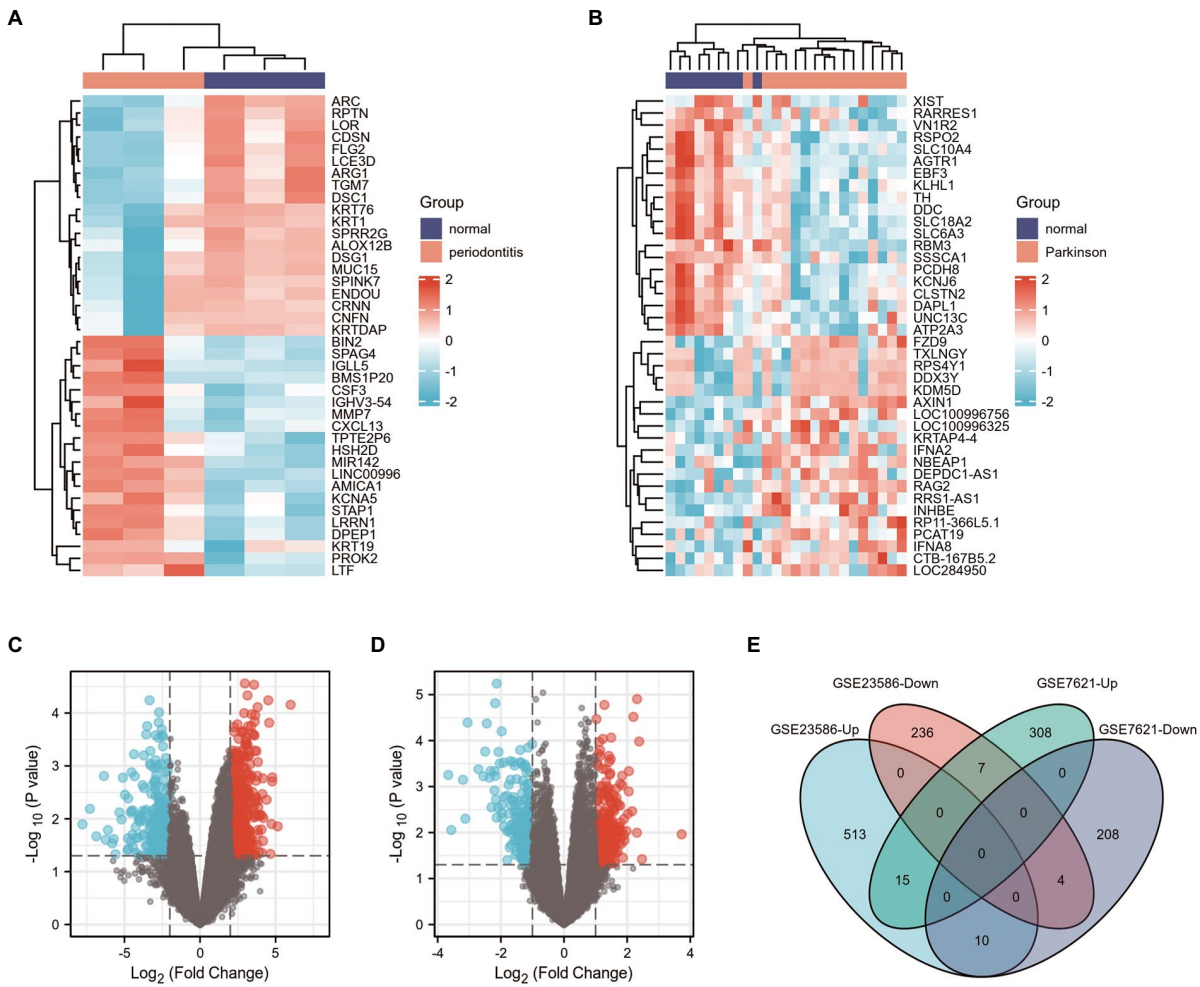
Screening of DEGs in GSE23586 and GSE7621 datasets. **(A)** Heatmap of DEGs between the normal sample and periodontitis sample in GSE23586 dataset. **(B)** Heatmap of DEGs between the normal sample and Parkinson’s disease sample in GSE7621 dataset. Red rectangles represent high expression, and blue rectangles represent low expression. **(C)** Volcano plot of DEGs between the normal sample and periodontitis sample in GSE23586 dataset. **(D)** Volcano plot of DEGs between the normal sample and Parkinson’s disease sample in GSE7621 dataset. Red plots represent upregulated genes, the black plots represent nonsignificant genes, and the blue plots represent downregulated genes. **(E)** Venn diagram of common DEGs from GSE23586 (|logFC| >2, and *p* < 0.05) and GSE7621 (|logFC| >1, and *p* < 0.05) dataset.

First, 785 DEGs were identified from healthy samples and periodontitis samples in the GSE23586 dataset, including 538 UP genes and 247 DOWN genes. Heat maps and volcano plots were used to visualize the DEGs (Figures 2A,C) using the|logFC| > 2 criterion with *p* < 0.05. However, 552 DEGs between normal and PD samples, including 330 UP genes and 222 DOWN genes, were from the GSE7621 dataset using|logFC| > 1 criterion with *p* < 0.05 (Figures 2B,D).

Venn diagrams of the DEGs in the GSE23586 and GSE7621 datasets are presented in Figure 2E. As seen, there were 15 common UP genes and four common DOWN genes. Details of these common genes are presented in Table 1.

**Table 1:** Common upregulated and downregulated DEGs in GSE23586 and GSE7621.

Gene symbol	*p*-value		Fold change		Gene title
	GSE23586-Up	GSE7621-Up	GSE23586-Up	GSE7621-Up	
*Up regulated genes*					
KCNA5	8.36E-03	1.62E-02	3.972	1.377	Potassium Voltage-Gated Channel Subfamily A Member 5
RP11-359E8.5	1.54E-03	4.38E-02	3.802	1.195	Long non-coding RNA
TNIP3	3.05E-02	2.26E-02	3.771	1.517	TNFAIP3 Interacting Protein 3
TSPAN1	9.50E-03	1.38E-02	3.571	1.103	Tetraspanin 1
CST1	5.15E-03	2.54E-02	3.183	1.214	Cystatin SN
CXCR4	1.84E-02	1.13E-02	3.004	1.114	C-X-C Motif Chemokine Receptor 4
NKX2-1	1.16E-02	5.28E-03	2.937	1.066	NK2 Homeobox 1
NLRC4	2.50E-03	5.39E-03	2.852	1.527	NLR Family CARD Domain Containing 4
IGJ	3.65E-02	4.54E-02	2.845	1.600	Joining Chain of Multimeric IgA And IgM
FW339973	2.55E-02	1.84E-02	2.828	1.499	FW339973.1
LOC101928896	2.80E-02	2.21E-02	2.405	1.051	Long non-coding RNA
DEFA5	3.09E-02	4.22E-02	2.180	1.144	Defensin Alpha 5
IL7R	2.06E-02	1.91E-02	2.149	1.267	Interleukin 7 Receptor
TRAF3IP3	6.82E-03	1.19E-02	2.132	1.189	TRAF3 Interacting Protein 3
HRC	3.89E-02	8.50E-03	2.030	1.302	Histidine Rich Calcium-Binding Protein
*Down regulated genes*					
DAPL1	1.19E-02	2.82E-03	−2.227	−2.296	Death-Associated Protein Like 1
ZNF208	2.91E-02	8.78E-03	−2.263	−1.370	Zinc Finger Protein 208
RDH12	2.33E-02	5.41E-03	−4.774	−1.319	Retinol Dehydrogenase 12
BPIFC	7.67E-03	4.65E-02	−4.590	−1.081	BPI Fold Containing Family C

### Functional and pathway enrichment of DEGs

**Figure 3:**
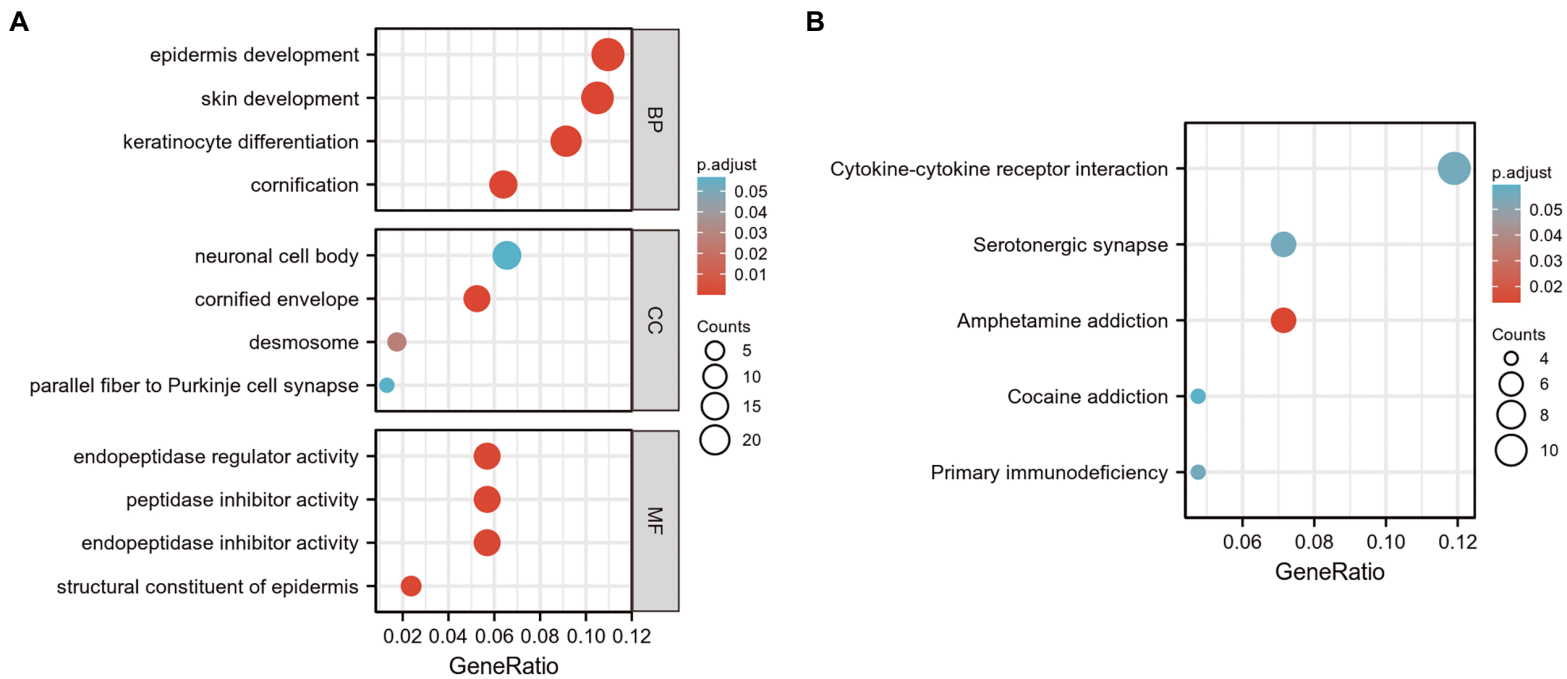
GO and KEGG analysis of DEGs in GSE23586 and GSE7621 datasets. **(A)** GO function enrichment analysis of DEGs in GSE23586 and GSE7621 dataset. GO, Gene Ontology; MF, Molecular Function; BP, Biological Process; CC, Cellular Components; DEGs, differentially expressed genes. **(B)** Enrichment analysis of the KEGG signaling pathway of DEGs in GSE23586 and GSE7621 dataset.

In the present study, 39 GO terms were identified, including 23 biological process (BP) terms, 4 cellular component (CC) terms, and 12 molecular function (MF) terms (adjusted *p*-value, *P*. adj. <0.1 & *q*-value <0.2). The top enriched functional category terms were are shown in Figure 3A and are presented in Table 2. The enriched GO terms included cornification, keratinocyte differentiation, skin development, and epidermis development in the BP category; cornified envelope, desmosome, neuronal cell body, and parallel fiber to Purkinje cell synapse in the CC category; and structural constituent of the epidermis, endopeptidase inhibitor activity, peptidase inhibitor activity, and endopeptidase regulator activity in the MF category.

**Table 2:** GO analysis of DEGs in GSE23586 and GSE7621.

Ontology	ID	Description	Gene Ratio	*p*-value	*p*. adjust
BP	GO:0070268	cornification	14/219	5.37e-11	1.43e-07
BP	GO:0030216	keratinocyte differentiation	20/219	5.71e-10	6.86e-07
BP	GO:0043588	skin development	23/219	9.10e-10	6.86e-07
BP	GO:0008544	epidermis development	24/219	1.26e-09	6.86e-07
CC	GO:0001533	cornified envelope	12/229	1.06e-11	3.10e-09
CC	GO:0030057	desmosome	4/229	1.85e-04	0.027
CC	GO:0043025	neuronal cell body	15/229	7.39e-04	0.057
CC	GO:0098688	parallel fiber to Purkinje cell synapse	3/229	7.74e-04	0.057
MF	GO:0030280	structural constituent of epidermis	5/211	9.02e-07	2.05e-04
MF	GO:0004866	endopeptidase inhibitor activity	12/211	1.29e-06	2.05e-04
MF	GO:0030414	peptidase inhibitor activity	12/211	1.95e-06	2.05e-04
MF	GO:0061135	endopeptidase regulator activity	12/211	1.95e-06	2.05e-04

*BP, biological processes; CC, cellular components; GO, Gene Ontology.*

Furthermore, seven enriched KEGG terms with *P*. adj. < 0.1 & *q*-value <0.2 were identified. The most enriched functional pathway terms are shown in Figure 3B and presented in Table 3. They included amphetamine addiction, primary immuno-deficiency, cytokine-cytokine receptor interaction, serotonergic synapse, and cocaine addiction.

**Table 3:** KEGG analysis of DEGs in GSE23586 and GSE7621.

ID	Description	Gene Ratio	*p*-value	*p*. adjust
hsa05031	Amphetamine addiction	6/84	7.51e-05	0.014
hsa05340	Primary immunodeficiency	4/84	6.14e-04	0.055
hsa04060	Cytokine-cytokine receptor interaction	10/84	9.15e-04	0.055
hsa04726	Serotonergic synapse	6/84	0.001	0.055
hsa05030	Cocaine addiction	4/84	0.002	0.059

### PPI network analysis of DEGs and hub gene selection

**Figure 4:**
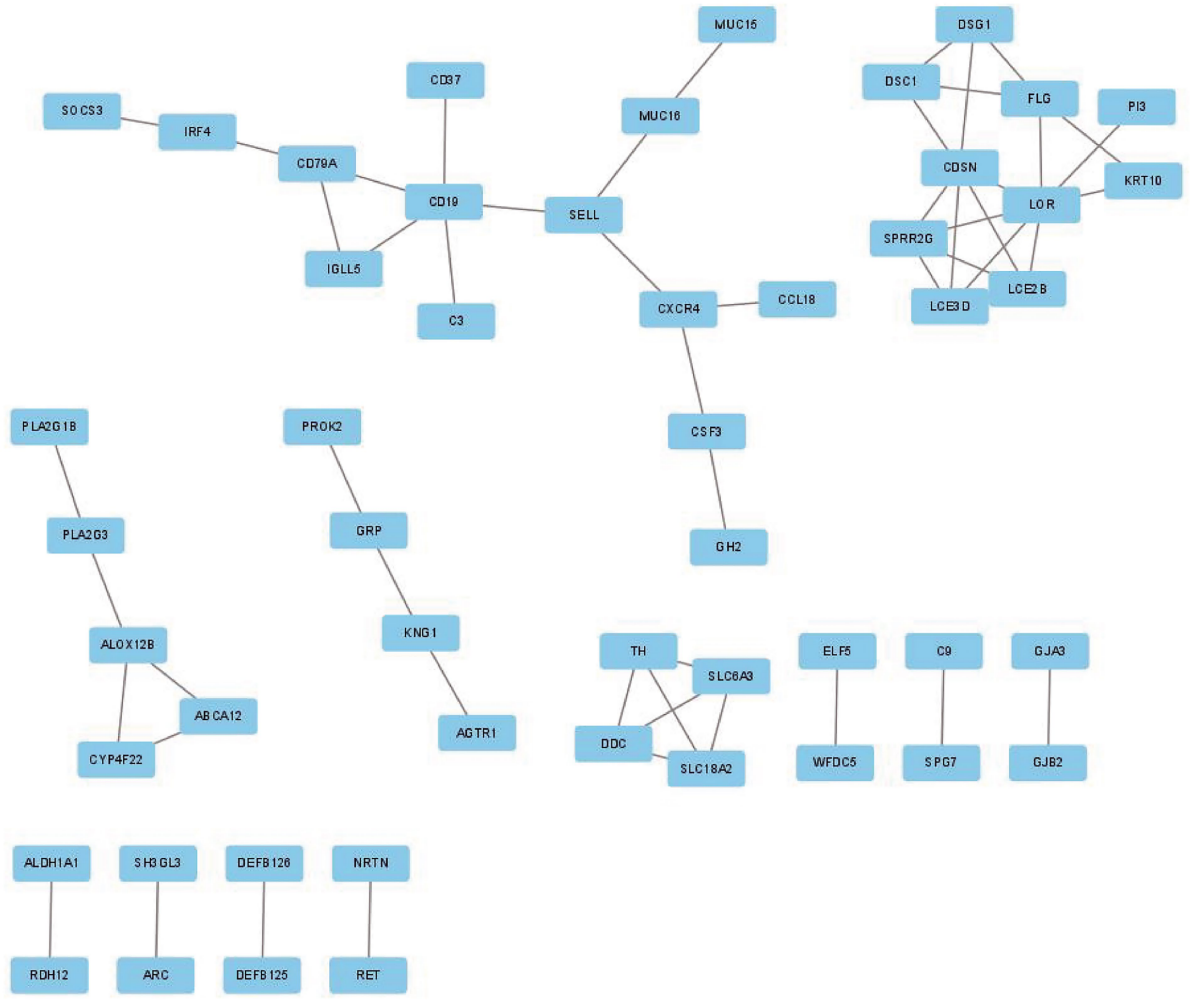
PPI network of DEGs in GSE23586 and GSE7621 dataset. PPI, protein–protein interaction; DEGs, differentially expressed genes.

**Figure 5:**
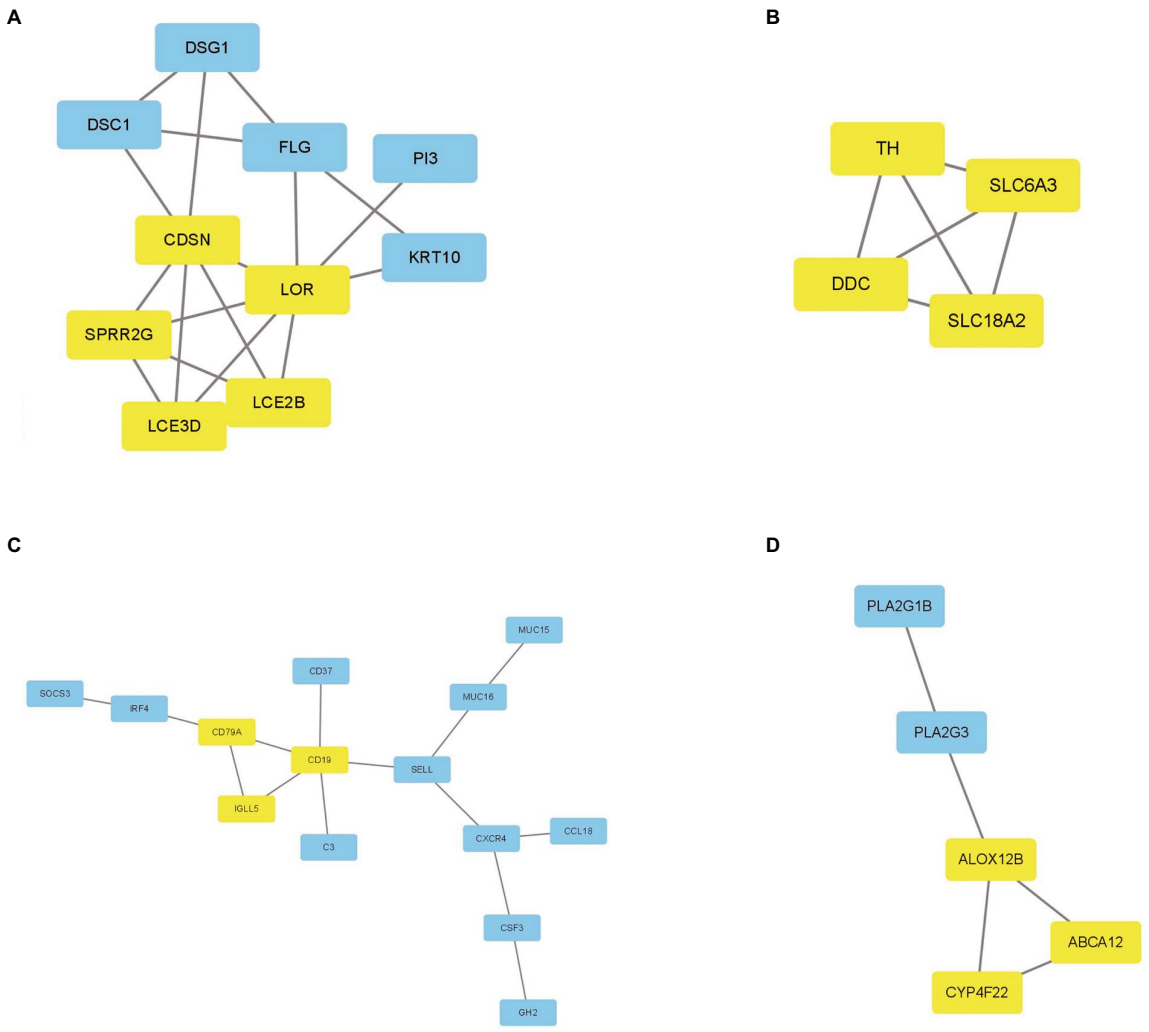
The key PPI network modules in GSE23586 and GSE7621 dataset. **(A–D)** Four key PPI network modules were performed by Cytoscape.

To investigate the association of the DEGs, PPI networks with an interaction score > 0.7 were analyzed in the STRING online database (Figure 4). We used the nodes to represent genes and the edges to represent connections between genes. Cytoscape was used to present the key PPI network modules. As shown in Figures 5A–D, four key modules were identified: late cornified envelope 2B (*LCE2B*), late cornified envelope 3D (*LCE3D*), corneodesmosin (*CDSN*), small proline-rich protein 2G (*SPRR2G*), loricrin cornified envelope precursor protein (*LOR*), tyrosine hydroxylase (*TH*), Dopa decarboxylase (*DDC*), solute carrier family 6 member 3 (*SLC6A3*), solute carrier family 18 member A2 (*SLC18A2*), arachidonate 12-lipoxygenase 12R type (*ALOX12B*), ATP-binding cassette subfamily A member 12 (*ABCA12*), cytochrome P450 family 4 subfamily F member 22 (*CYP4F22*), immunoglobulin lambda-like polypeptide 5 (*IGLL5*), CD19 molecule (*CD19*), and CD79a molecule (*CD79A*), which interacted with other proteins. These were the central nodes in the protein interaction network.

### Functional and pathway enrichment of hub genes

**Figure 6:**
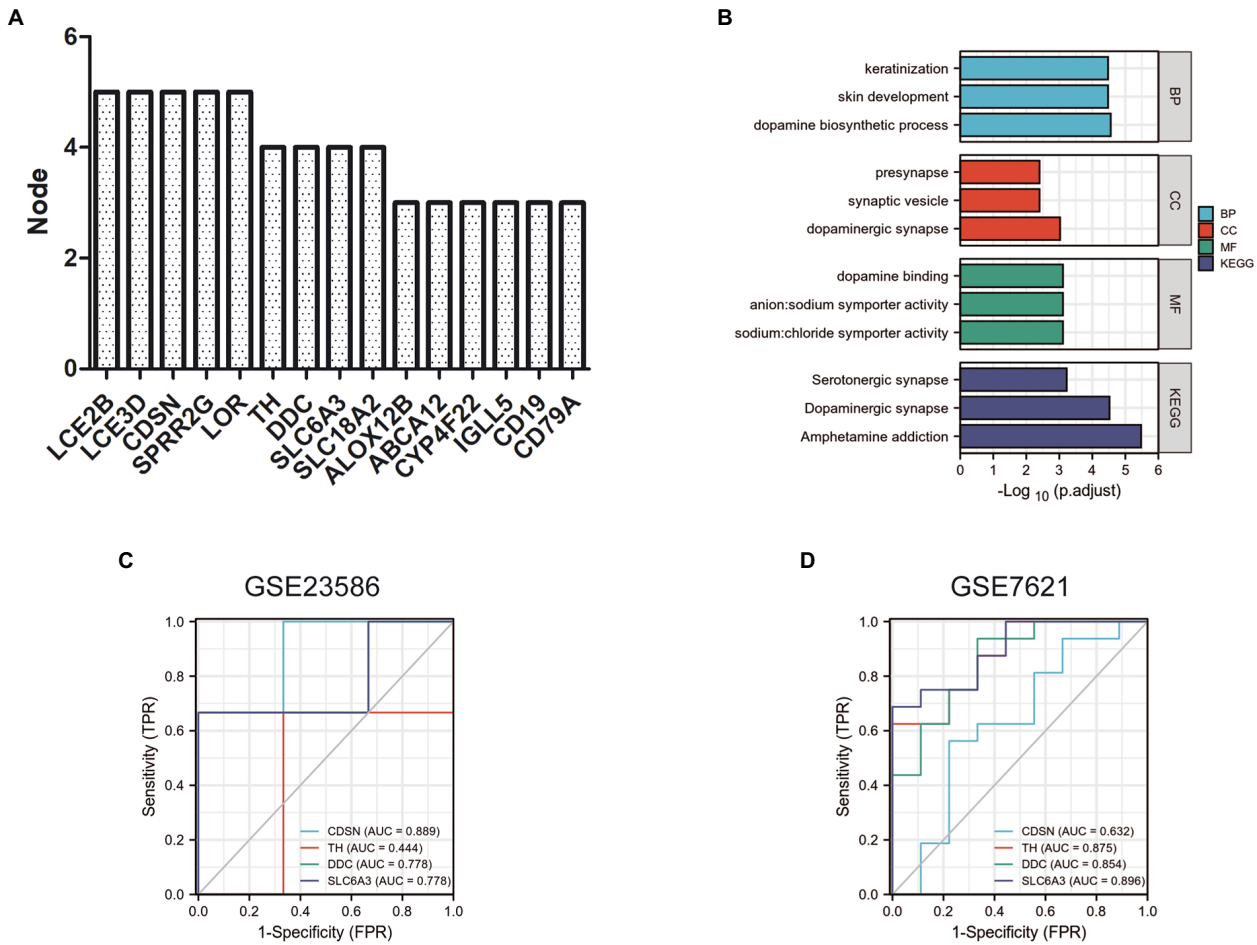
Functional enrichment analysis and ROC curve of hub genes. **(A)** Top 15 nodes of PPI networks of DEGs in GSE23586 and GSE7621 dataset. **(B)** GO and KEGG analysis of hub genes. Diagnostic value of 4 hub genes with ROC curves in GSE23586 dataset **(C)** and in GSE23586 dataset **(D)**. AUC area under the ROC curve.

The scores of these hub genes are shown in Figure 6A; Table 4. Functional enrichment analysis showed that the 15 hub genes were mainly linked to the dopamine biosynthetic process, skin development, and keratinization. There were three BP terms, as well as three CC terms (namely dopaminergic synapse, synaptic vesicle, and presynapse), and three MF terms (including sodium: chloride symporter activity, anion: sodium symporter activity, and dopamine binding; Figure 6B; Table 5).

**Table 4:** The hub genes and their functions.

Gene symbol	Description	Function
LCE2B	Late Cornified Envelope 2B	Epidermal differentiation
LCE3D	Late Cornified Envelope 3D	Nervous system development and Keratinization
CDSN	Corneodesmosin	Nervous system development and Keratinization
SPRR2G	Small Proline-Rich Protein 2G	Nervous system development and Keratinization
LOR	Loricrin Cornified Envelope Precursor Protein	Nervous system development and Keratinization
TH	Tyrosine Hydroxylase	Is involved in the conversion of tyrosine to dopamine.
DDC	Dopa Decarboxylase	Catalyzes the decarboxylation of L-3,4-dihydroxyphenylalanine (DOPA) to dopamine, L-5-hydroxytryptophan to serotonin and L-tryptophan to tryptamine.
SLC6A3	Solute Carrier Family 6 Member 3	a dopamine transporter
SLC18A2	Solute Carrier Family 18 Member A2	Transports amine neurotransmitters into synaptic vesicles.
ALOX12B	Arachidonate 12-Lipoxygenase, 12R Type	Arachidonic acid metabolism and Metabolism
ABCA12	ATP-Binding Cassette Subfamily A Member 12	A member of the superfamily of ATP-binding cassette (ABC) transporters.
CYP4F22	Cytochrome P450 Family 4 Subfamily F Member 22	A member of the cytochrome P450 superfamily of enzymes.
IGLL5	Immunoglobulin Lambda-Like Polypeptide 5	One of the immunoglobulin lambda-like polypeptides
CD19	CD19 Molecule	Functions as coreceptor for the B-cell antigen receptor complex (BCR) on B-lymphocytes.
CD79A	CD79a Molecule	Required in cooperation with CD79B for initiation of the signal transduction cascade activated by binding of antigen to the B-cell antigen receptor complex (BCR) which leads to internalization of the complex, trafficking to late endosomes and antigen presentation.

**Table 5:** Functional enrichment analysis of hub genes.

Ontology	ID	Description	GeneRatio	*p*-value	*p*. adjust
BP	GO:0042416	Dopamine biosynthetic process	3/14	7.36e-08	2.77e-05
BP	GO:0043588	Skin development	6/14	3.18e-07	3.34e-05
BP	GO:0031424	Keratinization	5/14	4.36e-07	3.34e-05
CC	GO:0098691	Dopaminergic synapse	2/14	3.64e-05	9.45e-04
CC	GO:0008021	Synaptic vesicle	3/14	3.01e-04	0.004
CC	GO:0098793	Presynapse	4/14	3.12e-04	0.004
MF	GO:0015378	Sodium:chloride symporter activity	2/13	2.23e-05	7.74e-04
MF	GO:0015373	Anion:sodium symporter activity	2/13	3.87e-05	7.74e-04
MF	GO:0035240	Dopamine binding	2/13	3.87e-05	7.74e-04
KEGG	hsa05031	Amphetamine addiction	4/8	3.33e-07	3.33e-06
KEGG	hsa04728	Dopaminergic synapse	4/8	4.54e-06	3.03e-05
KEGG	hsa04726	Serotonergic synapse	3/8	1.50e-04	5.98e-04

*BP, biological processes; CC, cellular components; GO, Gene Ontology.*

Furthermore, these 15 hub genes were significantly enriched in three KEGG signaling pathways: amphetamine addiction, dopaminergic synapses, and serotonergic synapses (Figure 6B; Table 5).

### Diagnostic values of the hub genes

To verify the diagnostic value of the hub genes obtained from the above analysis, we randomly selected four hub genes for further study analyses. The ROC curves were used to calculate the corresponding area under the curve (AUC) of the hub gene expression levels in the GSE23586 and GSE23586 datasets. As shown in Figure 6C, the AUC for *CDSN*, *TH*, *DDC*, and *SLC6A3* in periodontitis patients and normal controls were 0.889 (95% confidence interval [CI], 0.581–1.000, *p* < 0.05), 0.444 (95% CI, 0.000–1.000; *p* < 0.05), 0.778 (95% CI, 0.291–1.000; *p* < 0.05), and 0.778 (95% CI, 0.291–1.000; *p* < 0.05), respectively. In addition, the AUCs from the ROC curve of the four hub genes in the GSE7621 dataset (between PD patients and normal controls) were 0.632 (95% CI, 0.377–0.887; *p* < 0.05), 0.875 (95% CI, 0.737–1.000; *p* < 0.05), 0.854 (95% CI, 0.696–1.000; *p* < 0.05), and 0.896 (95% CI, 0.774–1.000; *p* < 0.05) (Figure 6D).

## Discussion

Neurodegenerative diseases are a group of progressive diseases that affect the central nervous system (CNS); examples include AD, PD, and HD (Sengupta and Kayed, 2022). Inflammation is considered as one of the contributing factors to neurodegeneration; it plays a key role in the occurrence and progression of these injuries (Kaur et al., 2016). Periodontitis is an inflammatory disease, caused by oral biofilms in the supporting tissues of teeth, which can lead to systemic and chronic inflammatory (Alvarenga et al., 2021). It has been reported that chronic oral infections, such as periodontitis, might aggravate the neuroinflammation of HD (Coyago and Temiño, 2015). Moreover, some studies demonstrated the association between periodontitis and neuroinflammation (Sansores-España et al., 2021), cognitive decline (Wang et al., 2019). Periodontitis, which impairs the insulin signaling pathway and stimulates gliosis and neuroinflammation, is a risk factor for AD (Duan et al., 2022). Moreover, phosphoglycerol dihydroceramide (produced by the periodontal pathogen *Porphyromonas gingivalis*) promotes amyloidogenesis and hyperphosphorylation, and promotes the pathogenesis of AD (Yamada et al., 2020).

It is well known that the loss of dopaminergic neurons in the substantia nigra is the major cause of PD (Poewe et al., 2017). Recently, multiple lines of evidence have supported the association between PD and inflammatory processes (Joutsa et al., 2018). For example, abnormal activation of the NLRP3 inflammasome can upregulate the expression of inflammatory cytokines interleukin-1β (IL-1β) and IL-18, leading to pathological inflammation in PD (He et al., 2020). Therefore, given that periodontitis is a chronic systemic inflammation, its association with PD has attracted increasing attention from researchers. To explore causality in this bidirectional association, some researchers have used two-sample Mendelian randomization (MR) in European ancestry populations. However, the MR study found no bidirectional causal genetic predisposition between periodontitis and PD (Botelho et al., 2021). Surprisingly, some studies using population-based retrospectively matched cohorts have found that patients with periodontitis are at a higher risk of developing PD (Chen et al., 2017).

*P. gingivalis*, the most common bacterium responsible for chronic periodontitis, was recently found to play an important role in the pathophysiology of PD (Olsen et al., 2020). For example, oral administration of *P. gingivalis* not only found reduce dopaminergic neurons and increase activated microglia in the substantia nigra of mice, but it also induced an inflammatory response and promoted PD (Feng et al., 2020). In addition, there are other oral microbial infections that also play a key role in the development of PD lesions. For instance, the microbiota of patients with PD has been found to contain less Firmicutes (such as Faecalibacterium, Clostridium, and Blautia) and more Bacteroidetes (Bacteroides and Prevotella) than healthy controls (Boeri et al., 2021). The study aimed to investigate the association between neurodegenerative disorders and periodontitis.

In the present study, we obtained the common DEGs between periodontitis and PD from two GEO datasets, and performed GO and KEGG enrichment analyses. Our results suggest that the common DEGs were mainly enriched in nervous, immunity, and other related pathways, indicating that there may be a relationship between these two inflammation-related diseases. By constructing a PPI network, we identified 15 key hub genes. Through more in-depth enrichment analyses of these hub genes, our speculation was verified; that is, these genes were found to be mainly involved in PD-related pathways, such as dopamine, nervous, and immunity pathways. These results suggest that the identified hub genes may play a key role in the occurrence and progression of PD. To further verify whether these genes could serve as potential PD diagnostic or therapeutic markers, we evaluated the ROC diagnostic value of four genes (*TH*, *CDSN*, *DDC*, and *SLC6A3*), and the results showed that they have a high diagnostic value. It has been reported that most of midbrain dopamine (mDA) neurons is permanently lost by the clinical diagnosis of PD (Kishinevsky et al., 2018). Therefore, the development of predictive biomarkers is a key area of PD research. The hub genes screened in this study might become potential diagnostic or therapeutic biomarkers of PD in future.

Tyrosine hydroxylase (*TH*) is a key enzyme that controls the synthesis of catecholamines, including dopamine, and plays a key role in PD caused by the degeneration of the dopaminergic neurons (Nagatsu et al., 2019). It has been reported that surviving neurons in the brains of PD patients have significantly increased levels of TH phosphorylation (Shehadeh et al., 2019). Moreover, obesity-related stimuli upregulate TH levels in periodontal cells and tissues, indicating that *TH* may be a key pathogenic factor in obesity-induced periodontitis (Memmert et al., 2019). Corneodesmosin (*CDSN*) is primarily involved in the development of the nervous system and in keratinization. It has been reported that *CDSN* gene mutation causes scalp peeling and hypotrichosis simplex of the scalp (van der Velden et al., 2020). Dopa Decarboxylase (*DDC*) is a pyridoxal 5′-phosphate (PLP) enzyme involved in the biosynthesis of dopamine and serotonin, and is associated with PD (Daidone et al., 2012). In addition, *SLC6A3* is a risk factor for PD (Zhai et al., 2014). The *SLC6A3* genotype has a significant effect on fronto-striatal activation and performance in PD (Habak et al., 2014). These findings are consistent with the diagnostic value we assessed. Overall, *TH*, *DDC*, and *SLC6A3* were the hub genes obtained in our study, indicating that our results are verifiable and credible.

In summary, using a series of bioinformatics tools for gene expression profiling, we established PPI network between PD and periodontitis, and screened key candidate genes, including *CDSN*, *TH*, *DDC*, and *SLC6A3*. Simultaneously, we enriched signaling pathways in the molecular modulation network through bioinformatics analysis. Our findings suggest a close relationship between chronic periodontitis and PD. Our results provide prospective targets for the future diagnosis and treatment of PD patients with chronic periodontitis. Furthermore, subsequent *in vitro* and *in vivo* studies should be performed to validate our findings. Our results may guide future research on the molecular mechanisms underlying the relationship between PD and periodontitis, and may provide new potential targets for diagnosis and therapy.

## Data availability statement

The datasets presented in this study can be found in online repositories. The names of the repository/repositories and accession number (s) can be found in the article/Supplementary material.

## Author contributions

MH and CY designed this study. XW and MH were responsible for the interpretation of the results. LY and BW processed the data processing and performed the statistical analysis. XW and NS wrote and revised the manuscript. All authors contributed to the article and approved the submitted version.

## Funding

This research was supported by Department of Science and Technology of Jilin Province (grant nos. YDZJ202201ZYTS021, YDZJ202201ZYTS033, and 20200201398JC).

## Conflict of interest

The authors declare that the research was conducted in the absence of any commercial or financial relationships that could be construed as a potential conflict of interest.

## Publisher’s note

All claims expressed in this article are solely those of the authors and do not necessarily represent those of their affiliated organizations, or those of the publisher, the editors and the reviewers. Any product that may be evaluated in this article, or claim that may be made by its manufacturer, is not guaranteed or endorsed by the publisher.

## Footnotes

^1^ http://www.ncbi.nlm.nih.gov/geo

^2^ http://bioinformatics.psb.ugent.be/webtools/Venn/

^3^ http://string-db.org/
